# Evaluation of the Reliability and Validity of a Food Frequency Questionnaire Using Three-Day 24-Hour Dietary Recalls: A Study in Fujian, China

**DOI:** 10.3390/nu17142270

**Published:** 2025-07-09

**Authors:** Lu Cheng, Yuhang Chen, Zhijie Luo, Qingying Wang, Fengqin Zou, Yulan Lin

**Affiliations:** Department of Epidemiology and Health Statistics, Fujian Provincial Key Laboratory of Environment Factors and Cancer, School of Public Health, Fujian Medical University, Fuzhou 350122, China; lucheng@fjmu.edu.cn (L.C.); yuhangchen@fjmu.edu.cn (Y.C.); zhijieluo@fjmu.edu.cn (Z.L.); qingyingwang@fjmu.edu.cn (Q.W.); zfengq@fjmu.edu.cn (F.Z.)

**Keywords:** food frequency questionnaire, reliability, validity, China

## Abstract

**Objective:** This study aimed to evaluate the reliability and validity of a Food Frequency Questionnaire (FFQ) designed for use in epidemiological studies among populations in Fujian, China. **Methods:** From September to December 2023, adults aged 18 years and above residing in Fujian Province, southeastern China, were recruited via online survey promotion. Participants completed the FFQ twice with a one-month interval and also completed a 3-day 24 h dietary recall (3d-24HDR), covering two weekdays and one weekend day, during the same period. The reliability of the FFQ was assessed by comparing the average intake of food groups and nutrients between the two FFQs, using Spearman correlation coefficients, intraclass correlation coefficients (ICCs), and weighted Kappa coefficients based on tertile classification. Validity was evaluated by comparing the average intake values from the FFQs and the 3d-24HDR using similar methods, including Spearman correlation, weighted Kappa statistics, and Bland–Altman analysis. **Results:** A total of 152 participants completed two FFQs (for reliability assessment), and 142 participants completed the 3d-24HDR (for validity assessment). Spearman correlation coefficients for food group intake between the two FFQs ranged from 0.60 to 0.80, with ICCs ranging from 0.53 to 0.91. For energy and nutrient intake, Spearman coefficients ranged from 0.66 to 0.96, and ICCs ranged from 0.57 to 0.97. After tertile classification, less than 15% of participants were misclassified into distant categories. The weighted Kappa coefficients for food groups and nutrients ranged from 0.37 to 0.71 and 0.43 to 0.88, respectively. In comparison with the 3d-24HDR, Spearman correlations for food groups and nutrients ranged from 0.41 to 0.72 and 0.40 to 0.70, respectively. The proportion of participants classified into the same or adjacent tertile was 78.8–95.1%. Weighted Kappa coefficients and Bland–Altman plots indicated acceptable agreement between the FFQ and 3d-24HDR for most nutrients. **Conclusions:** The FFQ used in this study demonstrated good reliability and moderate-to-good validity. It is suitable for use in dietary assessment in gastric cancer epidemiological studies in Fujian, China.

## 1. Introduction

With global population aging and ongoing changes in lifestyle, chronic non-communicable diseases (NCDs) have become a major public health challenge worldwide. The incidence of hypertension, diabetes, cardiovascular diseases, and various cancers continues to rise, imposing a substantial burden on health systems and economies [1,2]. Numerous studies have shown that diet is a key modifiable factor in the development and progression of NCDs [3,4]. Dietary patterns are closely associated with a variety of chronic diseases; for example, high sodium and low potassium intake is linked to an increased risk of hypertension, while long-term consumption of high-calorie and high-fat diets is a well-established risk factor for type 2 diabetes and cardiovascular disease [5,6]. In addition, unhealthy dietary habits have been implicated in the etiology of several cancers, including colorectal, breast, and lung cancer [7]. Individual dietary intake not only directly affects nutritional status but also influences disease mechanisms by modulating nutrition-related biomarkers, such as exposure markers, functional markers, and markers of health or disease status [8]. Therefore, accurately capturing long-term dietary intake is essential in epidemiological research to elucidate the relationship between diet and chronic diseases. The assessment of food consumption serves as the initial step in evaluating the nutritional status of a population. Based on such data, the intake of specific nutrients can be estimated through appropriate computational and analytical methods, such as food composition databases and professional dietary analysis software. This enables subsequent investigations into the associations between nutrient intake and health outcomes.

However, dietary exposure is inherently difficult to measure due to individual variability in food types and intake levels, as well as intra-individual variability across days (e.g., weekdays versus weekends). As a result, individuals often struggle to recall the exact types and amounts of food consumed, and inaccurate dietary assessments may become a major barrier to understanding the role of diet in disease etiology [9,10].

Currently, common dietary assessment methods include Weighed Dietary Records (WDRs), 24 h Dietary Recalls (24HDRs), and Food Frequency Questionnaires (FFQs) [11]. Due to their practicality, low cost, and ability to capture habitual intake, FFQs are widely used to assess long-term dietary patterns and their associations with health outcomes [12,13]. However, the validity and reliability of FFQs are strongly influenced by cultural dietary differences and the questionnaire’s design. Therefore, whether newly developed or adapted from other populations, FFQs must undergo rigorous validation before being applied in specific populations [14].

The reliability of FFQs is typically evaluated through test–retest reliability, where the same FFQ is administered to the same population twice within a 2–4-week interval. The consistency of nutrient or food group intake between the two measurements is assessed using Pearson or Spearman correlation coefficients or an intraclass correlation coefficient (ICC) [15,16]. Validity is usually evaluated by comparing FFQ data with reference methods such as WDRs or multiple-day 24HDRs. Although WDRs are considered a gold standard, their accuracy may be affected by participants’ motivation, food knowledge, and cultural context. The burden of weighing and recording all foods may alter dietary behavior and reduce compliance. In contrast, 24HDRs, despite sharing similar sources of measurement error with FFQs, are better suited to populations with lower education levels or limited compliance, offering improved representativeness while reducing respondent burden [17].

Due to regional and cultural dietary differences, FFQs developed in other provinces of China are often not directly applicable to Fujian Province. Therefore, dietary surveys conducted in Fujian require a localized FFQ that reflects regional dietary characteristics to ensure the accuracy of dietary intake data. Based on years of experience in dietary assessment in Fujian, our team developed an FFQ tailored to the local population. The objective of this study was to systematically evaluate the reliability and validity of this FFQ, providing a robust tool for future research on diet–disease relationships in Fujian.

This study contributes both academically and practically by developing and validating an FFQ specifically adapted to the dietary patterns of adults in Fujian Province. Academically, it fills an important methodological gap in regional dietary assessment and provides evidence on the reliability and validity of FFQs in the Chinese context. Practically, the validated FFQ can be applied in future epidemiological studies on diet-related chronic diseases (e.g., gastric cancer) and serves as a useful tool for local nutrition surveillance and public health interventions targeting dietary behavior.

## 2. Methods

### 2.1. Study Participants and Design

From September to December 2023, participants were recruited via an online survey link. Eligible participants were adults aged 18 to 75 years who had resided in Fujian Province, China for at least 6 months within the past 12 months and who were considered generally healthy. Exclusion criteria included having major chronic diseases (e.g., cancer, diabetes, stroke) or reporting implausible daily energy intakes (i.e., >3600 kcal or <500 kcal for women; >4200 kcal or <600 kcal for men) [18].

Eligible participants first completed the Food Frequency Questionnaire (FFQ-1) based on their habitual dietary intake over the past 12 months and provided sociodemographic information. Approximately one month later, participants completed a second FFQ (FFQ-2) to assess test–retest reliability. Between the two FFQ assessments, participants were also asked to complete a three-day 24 h dietary recall (3d-24HDR), covering two weekdays and one weekend day, to evaluate the relative validity of the FFQ.

### 2.2. Questionnaire Instruments

Data were collected using a self-developed questionnaire designed by the research team, consisting of five parts: I. General information; II. Behavioral lifestyle; III. Dietary habit; IV. Physical activity; and V. The Food Frequency Questionnaire (FFQ).

The FFQ was administered twice: the first time at baseline (referred to as FFQ-1) and again one month later (FFQ-2) for test–retest reliability assessment. The content of FFQ-1 and FFQ-2 was identical, and the FFQ section of the full questionnaire is presented in Supplemental File S1.

#### 2.2.1. Sociodemographic Information

The following information was collected: name, gender, age, height, weight, ethnicity, place of birth, marital status, education level, occupation, average monthly household income, type of health insurance, perceived stress level, smoking status, alcohol and tea consumption, physical activity, history of chronic diseases, and family history of cancer.

#### 2.2.2. Food Frequency Questionnaire (FFQ)

The FFQ included 78 food items or food groups across 13 major categories: staple foods (*n* = 8), tubers (*n* = 3), preserved/grilled/fried foods (*n* = 4), eggs (*n* = 2), fresh meats (*n* = 5), seafood (*n* = 5), dairy products (*n* = 4), snacks and nuts (*n* = 4), beverages (*n* = 3), soy products (*n* = 6), fresh vegetables (*n* = 17), fresh fruits (*n* = 12), and dried foods (*n* = 5). Frequency options for each food item included (1) ≥4 times/day; (2) 2–3 times/day; (3) once/day; (4) 4–6 times/week; (5) 2–3 times/week; (6) once/week; (7) 1–3 times/month; (8) occasionally; and (9) never.

For analytical purposes, food items were further categorized into 16 groups based on differences in the glycemic index (GI) for staple foods and nutritional/biological distinctions between red and white meat. The 16 groups included refined rice products, wheat products, whole grains, root and tuber vegetables, processed meats, eggs, red meat, poultry, organ meats, seafood, dairy products, snacks and nuts, legumes/soy products, fruits, vegetables, and beverages.

### 2.3. Three-Day 24-Hour Dietary Recall (3d-24HDR)

The 3d-24HDR collected detailed information on all foods consumed in the past 24 h, including food items, time of consumption (e.g., breakfast, lunch, dinner, snacks, late-night meals), eating context (e.g., at home, at a friend’s house, in restaurants, takeout, at work), and portion size (measured in Chinese liang or milliliters). The first page of the questionnaire included instructions and examples. To assist participants in accurately estimating intake, colored food photographs and portion size reference guides were provided (Supplemental File S2).

### 2.4. Calculation of Food Group and Nutrient Intake

The daily intake (g/day) of each food item in the FFQ was calculated using the following formula:

Daily intake = average portion size × frequency weighting factor.

The average portion size was estimated based on 3d-24HDR data, and the frequency weighting factors are detailed in Table 1. In the 3d-24HDR, the daily intake of each food item (g/day) was calculated as the total intake over three days divided by 3. The intake for each food group was determined by summing the daily intakes of all items within that group, as defined in the food classification table (Supplementary Table S1), and reported in g/day or mL/day.

Nutrient intakes from both the FFQ and the 3d-24HDR were calculated using the Nutrition Calculation Software (version 2.8.0(k)) developed by the National Institute for Nutrition and Food Safety, Chinese Center for Disease Control and Prevention. Nutrient values were matched to the Chinese Food Composition Table (6th Edition).

### 2.5. Statistical Analysis

All data were entered using EpiData version 3.1 and analyzed with SPSS version 26.0 (IBM Corp., Armonk, NY, USA). Continuous variables such as age and body mass index (BMI) were described using means and standard deviations (SDs), while food group and nutrient intake from FFQ-1, FFQ-2, and 3d-24HDR were presented as medians (M) and interquartile ranges (IQRs). Categorical variables (e.g., sex, education level) were summarized as frequencies and percentages. The Wilcoxon rank-sum test was used to compare the average intake levels of food groups and nutrients between FFQ-1, FFQ-2, and 3d-24HDR.

To control for potential confounding by total energy intake, the residual method was used for energy adjustment. Specifically, linear regression was performed with the total intake of each food or nutrient as the dependent variable and total energy intake as the independent variable. The residuals (i.e., the difference between observed and predicted values) were then used in subsequent analyses [19].

#### 2.5.1. Reliability Analysis

Spearman’s correlation coefficient (SCC) and the intraclass correlation coefficient (ICC) were used to assess the consistency of both raw and energy-adjusted data. Food and nutrient intakes from the two FFQs were categorized into tertiles, and weighted Kappa coefficients were calculated to evaluate agreement between FFQ-1 and FFQ-2. Kappa values were interpreted as follows: <0.20, poor agreement; 0.20–0.40, fair; 0.40–0.60, moderate; >0.60, good agreement [20,21].

#### 2.5.2. Validity Analysis

To evaluate validity, the average of the medians from FFQ-1 and FFQ-2 (M_FFQ-1_ + M_FFQ-2_/2) was used as the representative FFQ value and compared with the median intake from the 3d-24HDR (M_3d-24HDRs_). Spearman’s correlation coefficients were used to assess the correlation between the two methods for both food groups and nutrient intakes. Tertile classification was also applied to energy, food group, and nutrient intakes obtained from both methods, and weighted Kappa coefficients were used to evaluate agreement in classification.

To further assess agreement in nutrient intake estimation, Bland–Altman plots were constructed. The *x*-axis represented the mean of the median intakes from the FFQ and 3d-24HDR $\frac{\left(\left(M_{FFQ-1}+M_{FFQ-2}\right)/2+M_{3d-24HDR}\right)}{2}$, and the *y*-axis represented the difference in their medians $\frac{\left(\left(M_{FFQ-1}+M_{FFQ-2}\right)/2+M_{3d-24HDR}\right)}{2}$. The mean difference and 95% limits of agreement (LOA) were calculated as 95% LOA = mean difference ± 1.96 × SD. All *p* values were based on two-sided tests, with statistical significance defined as *p* < 0.05.

### 2.6. Sample Size Calculation

The required sample size was calculated using the following formula: n = (Z_α_ + Z_β_)^2^σ^2^/d^2^, where σ is the standard deviation, and d is the allowable measurement error. Based on a previous study [22], we assumed σ^2^ = 1, α = 0.05, 1 − β = 0.8, Z_0.05_ = 1.96, Z_0.2_ = 0.84, yielding a minimum sample size of *n* = 110.

### 2.7. Ethical Considerations

This study adhered to the ethical principles outlined in the Declaration of Helsinki and was approved by the Ethics Committee of Fujian Medical University (Approval No. FJMU 2020[53]). All participants provided written informed consent after being fully informed of the study’s purpose, content, and procedures. Throughout the study, the principles of non-maleficence and confidentiality were strictly followed, and participants were free to withdraw at any time. All personal data were kept strictly confidential.

## 3. Results

### 3.1. General Characteristics of Participants

Between September and December 2023, a total of 152 eligible participants were recruited in Fujian Province. All participants completed two FFQs for reliability analysis, and 142 of them also completed the 3d-24HDR for validity analysis (Table 2).

Among the 152 participants who completed both FFQs, the mean age was 42.38 years (SD ± 11.92; range: 20–70 years), with 69 males (45.4%) and 83 females (54.6%). The mean BMI was 22.66 kg/m^2^ (SD ± 3.03), with 35 participants classified as overweight (23.0%) and 8 as obese (5.3%). More than half of the participants had a college degree or above (57.9%). Most were married (75.0%), and nearly half reported a household monthly income between RMB 6000 and 12,000 (Renminbi, Chinese Yuan, 48.0%).

Among the 142 participants who completed the 3d-24HDR, the mean age was 42.67 years (SD ± 11.87; range: 20–70 years), with 60 males (42.3%) and 82 females (57.7%). The mean BMI was 22.35 kg/m^2^ (SD ± 2.79), with 29 participants classified as overweight (20.4%) and 5 as obese (3.5%). A similar proportion (58.5%) had a college degree or above, and most were married (74.0%), with 49.3% reporting a monthly household income of RMB 6000–12,000.

### 3.2. Reliability Analysis

#### 3.2.1. Reliability of Food Group Intake from FFQs

A comparison of the median intakes of food groups from FFQ-1 and FFQ-2 is presented in Table 3. Significant differences in median intake were observed for refined rice, red meat, seafood, legumes/soy products, and total vegetables (all *p* < 0.05), with FFQ-1 reporting higher intakes than FFQ-2.

The SCC for unadjusted food group intakes ranged from 0.60 (processed meat) to 0.82 (poultry), with a median of 0.71. The ICC ranged from 0.53 (legumes/soy products) to 0.91 (whole grains), with a median of 0.65. After energy adjustment, SCCs ranged from 0.30 (whole grains) to 0.80 (poultry), with a median of 0.66; ICCs ranged from 0.39 (whole grains) to 0.78 (processed meat), with a median of 0.59.

Tertile classification and weighted Kappa analysis showed that 48.7% to 87.5% of participants were classified into the same group in FFQ-1 and FFQ-2, while 0.0% to 12.5% were classified into non-adjacent groups. Except for root/tuber vegetables (κw = 0.37) and legumes/soy products (κw = 0.38), all food group Kappa coefficients were above 0.40 (Supplementary Table S2).

#### 3.2.2. Reliability of Energy and Nutrient Intakes from FFQs

Table 4 summarizes the comparison and correlation of energy and nutrient median intakes between FFQ-1 and FFQ-2. Significant differences (*p* < 0.05) were observed in the median intake of energy, protein, dietary fiber, vitamin B2, calcium, and manganese, with higher values reported in FFQ-1.

Before energy adjustment, SCCs ranged from 0.66 (energy) to 0.96 (folate), with a median of 0.79; ICCs ranged from 0.57 (vitamin E) to 0.97 (folate), with a median of 0.76. After energy adjustment, SCCs ranged from 0.33 (iron) to 0.94 (folate), with a median of 0.61; ICCs ranged from 0.30 (iron) to 0.93 (folate), with a median of 0.57.

Tertile classification and weighted Kappa analysis showed that 53.9% to 75.7% of participants were placed in the same group, while 1.4% to 9.3% were placed in non-adjacent groups. All weighted Kappa values exceeded 0.40, with seven nutrients demonstrating substantial agreement (κw > 0.60), including folate, vitamin B2, vitamin B6, calcium, magnesium, copper, and manganese (Supplementary Table S3).

### 3.3. Validity Analysis

#### 3.3.1. Validity of Food Group Intake

Table 5 presents the comparison of the median food group intake between the average of the two FFQs and the 3d-24HDR. Among the six food groups with significant differences (*p* < 0.05), only wheat-based products showed a lower intake in the FFQ compared to the 3d-24HDR; the other five food groups had higher median intakes in the FFQ.

Before energy adjustment, SCCs ranged from 0.41 (eggs) to 0.72 (total fruits), with a median of 0.57. Except for eggs, red meat, and total vegetables, all food groups had SCCs > 0.50. After energy adjustment, SCCs ranged from 0.27 (processed meat) to 0.66 (total fruits), with a median of 0.51.

Tertile agreement analysis showed that 46.5% to 73.2% of participants were classified into the same group across the FFQ and 3d-24HDR, and 4.9% to 21.2% were in non-adjacent groups. Eight food groups (50.0%) had weighted Kappa coefficients between 0.20 and 0.40, while the remaining groups had values ranging from 0.42 to 0.55 (Supplementary Table S4).

#### 3.3.2. Validity of Energy and Nutrient Intake

Table 6 compares the median intakes of energy and nutrients between the two FFQs and the 3d-24HDR. Among the 13 nutrients with significant differences (*p* < 0.05), the FFQ overestimated all except copper.

Before energy adjustment, SCCs ranged from 0.40 (selenium) to 0.70 (protein), with a median of 0.55. Except for fat, folate, vitamin B3, vitamin B6, selenium, and manganese, all nutrients had SCCs > 0.50. After energy adjustment, SCCs ranged from 0.25 (fat) to 0.63 (calcium), with a median of 0.50.

The Bland–Altman analysis was used to assess agreement between the FFQ and 3d-24HDR for nutrient intake (Supplementary Table S5). On average, the FFQ underestimated dietary fiber, vitamin B1, vitamin B6, vitamin E, calcium, copper, and energy intake (25.0%) and overestimated 11 other nutrients including protein, fat, and folate (45.8%).

**Figure 1:**
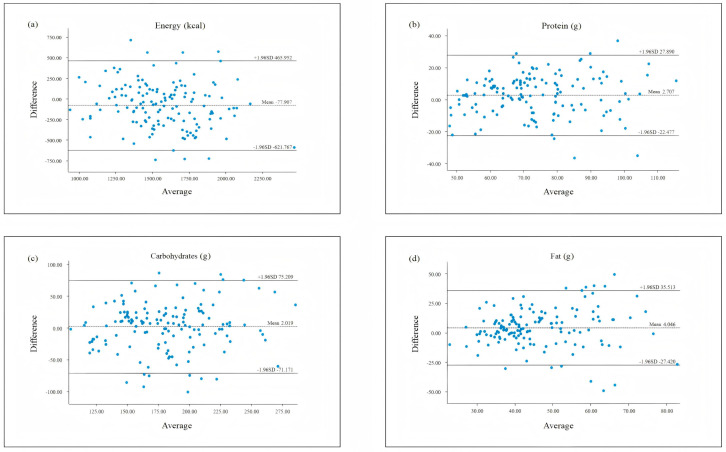
Bland–Altman scatter plots comparing nutrient intake assessed by the FFQ and 3d-24HDR. (**a**) Energy; (**b**) protein; (**c**) carbohydrates; (**d**) fat.

Figure 1 presents Bland–Altman plots for total energy and the three major macronutrients (protein, carbohydrate, and fat). The *x*-axis represents the average of median intake values from both methods, and the *y*-axis shows the difference between the two. The central line indicates the mean difference, and the upper and lower lines represent the 95% limits of agreement. The scatter plots showed that most points fell within the 95% limit, indicating acceptable agreement.

## 4. Discussion

This study aimed to evaluate the reliability and validity of a newly developed FFQ by our research team. Reliability was assessed by comparing the consistency between two FFQs administered one month apart, while validity was evaluated by comparing the FFQ results with dietary data collected via a 3d-24HDR. The findings indicated that the FFQ demonstrated moderate-to-good reliability and validity in assessing dietary intake among populations in Fujian Province, making it a suitable tool for obtaining accurate and effective dietary data in this region.

### 4.1. Reliability Evaluation of the FFQ

A comparison of median food group and nutrient intakes between FFQ-1 and FFQ-2 revealed that most differences were not statistically significant. Only 31.2% of food groups and 25.0% of nutrients showed significant differences, with FFQ-1 reporting higher median intakes than FFQ-2. This result aligns with findings from previous reliability studies [23]. One possible explanation is that respondents became more familiar with the questionnaire items by the time of the second administration and had improved portion size estimation after completing the 3d-24HDR, leading to more accurate responses in FFQ-2.

Reliability was further evaluated using the SCC and ICC based on both unadjusted and energy-adjusted data. The results showed that all SCCs and ICCs exceeded 0.50, indicating good reproducibility. Although no universal threshold exists for FFQ reliability, an SCC or ICC greater than 0.50 is generally considered acceptable for dietary intake assessment tools [24].

Interestingly, nutrients had higher SCC and ICC values than food groups. This may be due to greater variability in the types and quantities of foods consumed, whereas nutrient intakes are derived from multiple food sources, reducing individual-level variation [25]. In addition, the aggregation of food group data into nutrient values may mitigate random measurement errors, improving reliability estimates.

A meta-analysis of 30 FFQ validation studies in Chinese adults reported average SCCs and ICCs of 0.53 and 0.44 for food groups and 0.55 and 0.58 for nutrients, respectively [26]. Compared to these values, our study showed slightly higher reliability coefficients, possibly due to the shorter time interval between the two FFQ administrations. Prior FFQ reliability studies used intervals ranging from 1 week [27], 2 weeks [28,29], and 4 weeks to 1 month [30,31,32,33,34], to 3 months [35], 9 months [36], 1 year [37,38], and 2.7 years [39]. Shorter intervals typically yield higher reliability due to memory effects, where participants recall previous responses and repeat them, thus potentially inflating consistency [40]. Conversely, long intervals may result in genuine changes in dietary behavior, reducing FFQ reproducibility [41]. For instance, a study in Hubei Province using a 1-month interval reported SCCs of 0.84 for food groups and 0.85 for nutrients [42], which were higher than those in our study, likely due to the more homogeneous population of university students compared to the general adult sample in this study.

Tertile classification and weighted Kappa analysis further assessed reliability. For food groups, the proportion of participants classified into the same, adjacent, and opposite tertiles ranged from 48.7–87.5%, 12.5–46.7%, and 0.0–12.5%, respectively. For nutrients, the corresponding proportions were 53.9–75.7%, 20.4–41.5%, and 1.4–9.3%. These distributions are consistent with findings from other reliability studies [43,44]. For example, a Swiss FFQ reliability study reported 40.0–77.5% in the same tertile, 20.0–55.0% in adjacent tertiles, and 0–10.0% in opposite tertiles.

### 4.2. Validity Evaluation of the FFQ

#### 4.2.1. Choice of Validation Method

Dietary records (DRs) are considered the gold standard for dietary validation, as they minimize correlated errors with FFQs and do not rely on memory. However, they require high participant compliance and literacy, limiting their feasibility in general populations [45,46]. The 24HDR, despite sharing some errors with FFQs (e.g., recall and portion size biases), is widely used due to its feasibility and lower respondent burden [47,48]. In fact, 75% of validation studies reviewed by Cade et al. used 24HDR as the reference method [41]. Given that 40.6% of participants in our study had only a primary-level education or below, 24HDR was deemed more appropriate.

#### 4.2.2. FFQ Validity Analysis

A comparison of median food group and nutrient intakes between FFQs and 3d-24HDR showed that 37.5% of food groups and 54.2% of nutrients differed significantly. Among those with significant differences, FFQ median values were generally higher than those from 3d-24HDR, consistent with other studies [49,50]. The observed differences in intake estimates may be partly due to the differing timeframes of the two methods; the FFQ assesses habitual dietary intake over 12 months, whereas the 3-day 24HDR captures only short-term intake, which may omit infrequently consumed or seasonal foods (e.g., snacks, holiday meals). The discrepancy in the reference period between the FFQ (12 months) and the 3-day 24HDR is a recognized limitation in dietary validation studies. While it is true that an individual’s food intake over a full year encompasses a far wider variety of items than can be captured over just three days, the use of short-term recalls as a reference method is widely accepted in nutritional epidemiology. The primary objective of FFQ validation is not to precisely match absolute intake but to evaluate whether the FFQ can reasonably estimate the usual intake and accurately rank individuals according to their dietary intake levels. Numerous validation studies, both in China (e.g., the China Health and Nutrition Survey [CHNS]) and internationally (e.g., the National Health and Nutrition Examination Survey [NHANES]), have adopted similar validation protocols using two or three 24 h dietary recalls to assess the relative validity of FFQs. This approach is particularly common when more intensive reference methods, such as weighed food records, are impractical due to participant burden, low compliance, or limited literacy levels [51,52]. In our study, we followed this widely used approach while fully acknowledging its limitations. This methodology provides a practical balance between precision and feasibility in large-scale or community-based research settings. We have also included both correlation coefficients and agreement analyses (e.g., weighted Kappa and Bland–Altman plots) to strengthen the evaluation of relative validity despite the time frame discrepancy.

The SCCs for food group validity ranged from 0.41 to 0.72 (median: 0.57), while those for nutrient intake ranged from 0.40 to 0.70 (median: 0.55). According to Cui et al. [24], FFQ validity coefficients between 0.40 and 0.70 are acceptable when 24HDR is used as a reference method, suggesting that our FFQ demonstrated reasonable agreement.

As no dietary assessment tool is entirely free from measurement error [19], we applied energy adjustment using the residual method to reduce potential confounding and correlated errors. After adjustment, most validity coefficients declined, indicating that systematic measurement error rather than energy intake per se accounted for much of the variance [53]. Similar trends have been reported in the Shanghai Men’s Health Study and studies of Moroccan adults [33,54].

Additionally, Bland–Altman plots were used to further assess agreement between the FFQ and the 3d-24HDR [55]. These plots revealed relatively small discrepancies at lower intake levels and wider dispersion at higher intake levels, a pattern commonly observed in previous studies [56,57]. The majority of data points fell within the 95% limits of agreement and showed no systematic bias, indicating an overall acceptable level of agreement between the two methods.

### 4.3. Limitations

This study has several limitations. First, both FFQ and 3d-24HDR are self-reported tools and may be subject to recall bias. To minimize confounding by total energy intake, we applied energy adjustment in all reliability and validity analyses. Second, only one 3-day dietary recall was collected, limiting our ability to account for seasonal variation. Third, the FFQ and its validation were restricted to adults aged 18 and older in Fujian, excluding children and adolescents. Therefore, caution is needed when applying this FFQ to other populations, and further validation is required.

## 5. Conclusions

This study comprehensively assessed the reliability and validity of a newly developed FFQ for use in Fujian Province. All reliability coefficients exceeded 0.50, and weighted Kappa values indicated moderate-to-good consistency. Validity coefficients (based on 3d-24HDR) ranged from 0.40 to 0.72, and both weighted Kappa statistics and Bland–Altman plots demonstrated acceptable agreement. The FFQ showed good reliability and reasonable validity, making it suitable for dietary assessment and dietary pattern research among adults in Fujian. Compared with findings from recent validation studies conducted both in China and internationally, the FFQ developed in this study demonstrated comparable or superior performance in terms of reliability and validity, supporting its utility for dietary assessment in future epidemiological research. These findings provide a solid foundation for future investigations into diet–disease relationships.

## Figures and Tables

**Table 1:** Food frequency weighting coefficient.

Frequency	Rarely/Never	1–3 Times/Month	1 Time/Week	2–3 Times/Week	4–6 Times/week	1 Time/Day	2–3 Times/Day	4 Times/Day
Weight	0.00	0.07	0.14	0.36	0.71	1.00	2.50	4.00

**Table 2:** General demographic characteristics of the study population.

Variables	Reliability Study (N = 152)	Validity Study (N = 142)
Age (years), mean ± std	42.38 ± 11.92	42.67 ± 11.87
Age group (years)		
<30	19 (12.5)	17 (12.0)
30–40	55 (36.2)	50 (35.2)
>40	78 (51.3)	75 (52.8)
Sex		
Male	69 (45.4)	60 (42.3)
Female	83 (54.6)	82 (57.7)
BMI, kg/m^2^, mean ± std	22.66 ± 3.03	22.35 ± 2.79
BMI group (kg/m^2^)		
<25.0	109 (71.7)	108 (76.1)
25.0–29.9	35 (23.0)	29 (20.4)
≥30.0	8 (5.3)	5 (3.5)
Education level		
Primary school or below	18 (11.8)	18 (12.7)
Middle/high school	46 (30.3)	41 (28.9)
College or above	88 (57.9)	83 (58.5)
Marital status		
Unmarried	34 (22.4)	31 (21.8)
Married	114 (75.0)	107 (75.4)
Divorced/Widowed	4 (2.6)	4 (2.8)
Average monthly household income (RMB)		
<6000	45 (29.6)	44 (31.0)
6000–12,000	73 (48.0)	70 (49.3)
>12,000	34 (22.4)	28 (19.7)
Smoking		
Yes	29 (19.1)	23 (16.2)
No	123 (80.9)	119 (83.8)
Alcohol drinking		
Yes	49 (32.2)	43 (30.3)
No	103 (67.8)	99 (69.7)
History of chronic disease		
Yes	20 (13.2)	19 (13.4)
No	132 (86.8)	123 (86.6)
Family cancer history		
Yes	37 (24.3)	35 (24.6)
No	116 (76.3)	107 (75.4)

**Table 3:** Comparison and correlation analysis of food group intakes between FFQ-1 and FFQ-2.

Food (g/d)	FFQ-1	FFQ-2	*p* ^a^	SCC ^b^		ICC ^c^	
	M (P25–P75)	M (P25–P75)		Crude	Adjusted ^d^	Crude	Adjusted ^d^
Refined rice and related products	325.00 (185.00–437.50)	274.25 (185.50–437.50)	0.043	0.63 *	0.60 *	0.57	0.55
Wheat products (e.g., noodles, bread)	93.60 (47.20–141.70)	80.70 (37.80–120.58)	0.135	0.73 *	0.69 *	0.67	0.64
Whole grains	0.00 (0.00–10.85)	0.00 (0.00–10.50)	0.261	0.80 *	0.30 *	0.91	0.39
Root and tuber vegetables	14.00 (0.00–36.00)	7.00 (0.00–24.50)	0.265	0.67 *	0.66 *	0.55	0.53
Processed meat	0.00 (0.00–11.20)	0.00 (0.00–5.60)	0.447	0.60 *	0.58 *	0.80	0.78
Eggs	50.00 (35.50–50.00)	40.40 (35.50–50.00)	0.335	0.69 *	0.59 *	0.60	0.50
Red meat	125.00 (88.75–125.00)	88.75 (45.00–125.00)	0.045	0.63 *	0.46 *	0.61	0.44
Poultry	36.00 (0.00–71.00)	14.00 (0.00–36.00)	0.201	0.82 *	0.80 *	0.65	0.63
Organ meat	0.00 (0.00–0.00)	0.00 (0.00–5.60)	0.209	0.72 *	0.64 *	0.78	0.70
Seafood	85.00 (43.20–120.68)	56.63 (28.35–87.35)	<0.001	0.77 *	0.72 *	0.70	0.64
Dairy products	90.00 (90.00–177.50)	64.35 (17.50–169.70)	0.549	0.75 *	0.78 *	0.71	0.75
Snacks and nuts	6.30 (0.00–28.05)	5.60 (0.00–14.39)	0.828	0.67 *	0.64 *	0.65	0.62
Soybeans and soybean products	54.40 (26.89–98.85)	31.50 (15.75–52.50)	<0.001	0.69 *	0.67 *	0.53	0.54
Vegetables	297.91 (222.59–388.43)	256.08 (191.89–321.70)	<0.001	0.73 *	0.70 *	0.68	0.64
Fruits	107.09 (54.30–150.83)	107.09 (54.30–150.83)	0.338	0.73 *	0.65 *	0.64	0.56
Beverages	0.00 (0.00–42.00)	0.00 (0.00–35.00)	0.464	0.70 *	0.69 *	0.56	0.54

^a^ Wilcoxon rank-sum test; ^b^ SCC, Spearman’s rank correlation coefficient; ^c^ ICC, intraclass correlation coefficient; ^d^ energy adjustment was performed using the residual method; * *p* < 0.05.

**Table 4:** Comparison and correlation analysis of nutrient intakes between FFQ-1 and FFQ-2.

Nutrients	FFQ-1	FFQ-2	*p* ^a^	SCC ^b^		ICC ^c^	
	M (P25–P75)	M (P25–P75)		Crude	Adjusted ^d^	Crude	Adjusted ^d^
Energy (kcal)	1572.04 (1426.76–1757.33)	1469.67 (1222.87–1734.55)	0.004	0.66 *		0.67	
Protein (g)	75.44 (62.96–90.45)	68.64 (62.96–83.70)	0.046	0.77 *	0.41 *	0.75	0.38
Fat (g)	46.51 (38.26–58.60)	43.88 (37.21–53.31)	0.082	0.78 *	0.48 *	0.74	0.46
Carbohydrates (g)	180.93 (154.54–216.97)	175.33 (158.59–204.90)	0.369	0.83 *	0.67 *	0.88	0.65
Dietary fiber (g)	8.61 (6.70–10.98)	7.44 (6.30–9.21)	0.002	0.78 *	0.61 *	0.67	0.48
Cholesterol (mg)	507.84 (409.64–595.22)	518.50 (430.56–595.24)	0.629	0.77 *	0.65 *	0.74	0.63
Folate (mg)	104.61 (75.33–138.53)	105.76 (84.71–132.86)	0.844	0.96 *	0.94 *	0.97	0.93
Vitamin A (μg)	424.73 (350.54–553.15)	416.10 (349.33–516.51)	0.454	0.83 *	0.77 *	0.83	0.77
Vitamin B1 (mg)	0.64 (0.53–0.78)	0.62 (0.53–0.72)	0.166	0.69 *	0.34 *	0.71	0.31
Vitamin B2 (mg)	0.96 (0.77–1.13)	0.87 (0.72–1.06)	0.022	0.79 *	0.61 *	0.78	0.59
Vitamin B3 (mg)	17.69 (14.18–21.53)	16.31 (14.59–19.68)	0.133	0.77 *	0.48 *	0.74	0.46
Vitamin B6 (mg)	0.23 (0.15–0.29)	0.20 (0.15–0.26)	0.103	0.85 *	0.76 *	0.86	0.75
Vitamin C (mg)	84.06 (62.23–113.57)	81.38 (62.37–103.28)	0.237	0.81 *	0.75 *	0.76	0.70
Vitamin E (mg)	8.76 (6.57–10.97)	7.94 (6.54–9.65)	0.075	0.68 *	0.48 *	0.57	0.46
Calcium (mg)	553.26 (418.85–689.63)	482.75 (370.01–616.66)	0.014	0.85 *	0.75 *	0.78	0.68
Phosphorus (mg)	1072.02 (878.17–1221.47)	1028.42 (873.99–1146.06)	0.284	0.82 *	0.42 *	0.87	0.46
Potassium (mg)	1863.56 (1491.40–2190.27)	1825.29 (1512.76–2085.62)	0.178	0.84 *	0.60 *	0.82	0.58
Sodium (mg)	701.86 (567.13–858.22)	693.98 (605.33–802.53)	0.677	0.85 *	0.68 *	0.87	0.66
Magnesium (mg)	269.66 (218.81–308.70)	262.91 (215.92–308.67)	0.660	0.89 *	0.62 *	0.92	0.63
Iron (mg)	17.97 (15.03–21.07)	16.92 (15.47–20.26)	0.158	0.78 *	0.33 *	0.77	0.30
Zinc (mg)	13.45 (10.79–15.85)	13.45 (11.24–15.35)	0.850	0.78 *	0.51 *	0.78	0.49
Selenium (μg)	68.56 (52.33–80.24)	65.50 (54.28–75.34)	0.219	0.76 *	0.49 *	0.74	0.47
Copper (mg)	1.62 (1.28–2.15)	1.54 (1.24–1.93)	0.087	0.79 *	0.36 *	0.33	0.34
Manganese (mg)	4.18 (3.37–5.25)	3.89 (3.26–4.61)	0.020	0.76 *	0.55 *	0.73	0.54

^a^ Wilcoxon rank-sum test; ^b^ SCC, Spearman’s rank correlation coefficient; ^c^ ICC, intraclass correlation coefficient; ^d^ energy adjustment was performed using the residual method; * *p* < 0.05.

**Table 5:** Comparison and correlation analysis of food group intakes between FFQ and 3d-24HDR.

Food (g/d)	(FFQ-1 + FFQ-2)/2	3d-24HDR	*p* ^a^	SCC ^b^	
	M (P25–P75)	M (P25–P75)		Crude	Adjusted ^c^
Refined rice and related products	311.50 (202.75–437.50)	333.33 (245.83–400.00)	0.391	0.51 *	0.52 *
Wheat products (e.g., noodles, bread)	90.95 (43.09–126.68)	100.00 (56.67–163.33)	0.032	0.61 *	0.64 *
Whole grains	0.00 (0.00–10.68)	0.00 (0.00–34.17)	0.868	0.59 *	0.50 *
Root and tuber vegetables	14.00 (2.63–35.91)	16.67 (0.00–33.33)	0.079	0.58 *	0.48 *
Processed meat	0.00 (0.00–1.40)	0.00 (0.00–0.00)	0.069	0.64 *	0.27 *
Eggs	42.75 (34.00–50.00)	33.33 (16.67–50.00)	0.040	0.41 *	0.40 *
Red meat	106.88 (85.00–125.00)	130.00(80.00–180.00)	0.146	0.44 *	0.41 *
Poultry	18.00 (0.00–40.88)	16.67 (0.00–47.50)	0.207	0.54 *	0.56 *
Organ meat	0.00 (0.00–2.80)	0.00 (0.00–0.00)	0.071	0.57 *	0.55 *
Seafood	74.70 (42.43–102.41)	60.00 (24.58–103.33)	0.036	0.56 *	0.46 *
Dairy products	76.45 (8.75–177.50)	50.00 (0.00–133.33)	<0.001	0.63 *	0.62 *
Snacks and nuts	5.43 (0.88–21.54)	4.17 (0.00–30.83)	0.305	0.69 *	0.51 *
Soybeans and soybean products	48.11 (21.23–75.73)	35.84 (16.67–83.33)	0.117	0.55 *	0.53 *
Vegetables	275.83 (208.55–352.49)	266.67 (199.17–367.50)	0.740	0.45 *	0.41 *
Fruits	107.09 (50.15–150.83)	100.00 (33.33–141.25)	0.035	0.72 *	0.66 *
Beverages	0.00 (0.00–24.50)	0.00 (0.00–10.00)	0.008	0.54 *	0.50 *

^a^ Wilcoxon rank-sum test; ^b^ SCC, Spearman’s rank correlation coefficient; ^c^ ICC, intraclass correlation coefficient; * *p* < 0.05.

**Table 6:** Comparison and correlation analysis of nutrient intakes between FFQ and 3d-24HDR.

Nutrients	(M_FFQ-1_ + M_FFQ-2_)/2	3d-24HDR	*p* ^a^	SCC ^b^	
	M (P25–P75)	M (P25–P75)		Crude	Adjusted ^c^
Energy (kcal)	1519.94 (1344.21–1720.80)	1608.70 (1370.50–1841.75)	0.084	0.66 *	
Protein (g)	73.82 (64.82–84.34)	72.16 (60.33–82.75)	0.098	0.77 *	0.41 *
Fat (g)	44.30 (38.53–56.26)	44.07 (34.78–50.48)	0.008	0.78 *	0.48 *
Carbohydrates (g)	179.29 (158.47–211.41)	180.74 (145.83–207.38)	0.759	0.83 *	0.67 *
Dietary fiber (g)	8.10 (6.78–10.08)	8.70 (6.90–10.90)	0.111	0.78 *	0.61 *
Cholesterol (mg)	504.80 (406.97–595.31)	494.00 (395.5–658.5)	0.862	0.77 *	0.65 *
Folate (mg)	104.06 (79.10–134.01)	66.60 (49.68–97.38)	<0.001	0.96 *	0.94 *
Vitamin A (μg)	418.71 (344.00–523.23)	406.00 (333.25–509.50)	0.180	0.83 *	0.77 *
Vitamin B1 (mg)	0.63 (0.53–0.74)	0.61 (0.52–0.72)	0.241	0.69 *	0.34 *
Vitamin B2 (mg)	0.89 (0.75–1.09)	0.82 (0.67–1.02)	0.014	0.79 *	0.61 *
Vitamin B3 (mg)	17.18 (14.27–20.17)	16.57 (13.63–18.45)	0.034	0.77 *	0.48 *
Vitamin B6 (mg)	0.20 (0.15–0.27)	0.31 (0.20–0.42)	0.144	0.85 *	0.76 *
Vitamin C (mg)	81.00 (60.28–107.45)	58.60 (36.88–82.30)	<0.001	0.81 *	0.75 *
Vitamin E (mg)	8.51 (6.70–10.23)	8.60 (6.79–11.58)	0.192	0.68 *	0.48 *
Calcium (mg)	529.84 (394.52–645.50)	526.50 (414.75–710.00)	0.241	0.85 *	0.75 *
Phosphorus (mg)	1054.32 (888.11–1167.37)	973.00 (822.50–1148.00)	0.039	0.82 *	0.42 *
Potassium (mg)	1833.73 (1500.44–2128.20)	1708.50 (1349.25–2023.75)	0.045	0.84 *	0.60 *
Sodium (mg)	717.07 (568.00–841.36)	605.05 (475.78–772.75)	<0.001	0.85 *	0.68 *
Magnesium (mg)	267.24 (218.13–310.92)	241.00 (202–283.25)	0.660	0.89 *	0.62 *
Iron (mg)	17.57 (15.07–20.43)	17.00 (13.8–19.63)	0.158	0.78 *	0.33 *
Zinc (mg)	13.44 (11.14–15.86)	13.86 (10.85–16.33)	0.850	0.78 *	0.51 *
Selenium (μg)	66.21 (53.51–77.73)	54.57 (44.92–68.37)	0.219	0.76 *	0.49 *
Copper (mg)	1.58 (1.29–2.05)	1.80 (1.43–2.17)	0.087	0.79 *	0.36 *
Manganese (mg)	3.99 (3.31–5.00)	4.02 (3.43–4.81)	0.020	0.76 *	0.55 *

^a^ Wilcoxon rank-sum test; ^b^ SCC, Spearman’s rank correlation coefficient; ^c^ ICC, intraclass correlation coefficient; * *p* < 0.05.

## Data Availability

The data that support the findings of this study are available from the corresponding author, Yulan Lin, upon reasonable request. The data are not publicly available due to privacy and ethical restrictions.

## Supplementary Materials

The following supporting information can be downloaded at https://www.mdpi.com/article/10.3390/nu17142270/s1, Supplemental File S1: Questionnaire; Supplemental File S2: 24-hour dietary recall survey; Table S1: Food group table; Table S2: Agreement Analysis of Food Group Tertile Categorization Between FFQ-1 and FFQ-2; Table S3: Agreement Analysis of Nutrient Intake Tertile Categorization Between FFQ-1 and FFQ-2; Table S4: Agreement Analysis of Food Group Tertile Categorization Between FFQ and 24HDR; Table S5: Results of Bland-Altman analysis of nutrient intakes between FFQ and 3d-24HDR.
