# Supplementary material for: Evaluation of the Reliability and Validity of a Food Frequency Questionnaire Using Three-Day 24-Hour Dietary Recalls: A Study in Fujian, China
Source: Nutrients. 2025 Jul 9;17(14):2270. doi: 10.3390/nu17142270 (PMC12298913; doi:10.3390/nu17142270)
Supplement: Supplementary file 1 [file nutrients-17-02270-s001.zip › Supplemental File S1. Questionnaire.pdf]

No.：_______________ Case no.____________ Name:______

Appendix A: Full Questionnaire Content

The self-developed questionnaire used in this study consisted of the following five parts:

Part I. General information

Part II. Behavioral lifestyle

Part III. Dietary habit

Part IV. Physical activity

Part V. Food Frequency Questionnaire (FFQ)

For the validation of the FFQ's test－retest reliability, the Part V section was completed twice by participants:

FFQ-1: administered at baseline

FFQ-2: administered one month later

The content of FFQ-1 and FFQ-2 was identical; only the time of administration differed. To avoid redundancy, only one version (FFQ-1) is included in this appendix.

No.: ______________ Case no.____________ Name:_____

Health Status Questionnaire for Residents of Fujian Province

Name:____

Contact number: Landline:__________________________________

Cell phones: 1_________________

Cell phones: 2_________________

Address:__________ Province, ___________City, _____District/County

__________ Streets/Townships

No.：______________ Case no.____________ Name:_____

Informed consent

Dear participants:

In order to understand the health status and related living habits of the population and to promote disease prevention and control, researchers at Fujian Medical University plan to conduct a survey on the health status of the population. The survey will cover general conditions, living environment, diet, living habits and so on.

We sincerely hope that we can get strong support and sincere cooperation from you and your family! This survey requires the investigator to ask you face-to-face, which may cause you and your family a lot of inconvenience, please understand. The results of the survey will be kept strictly confidential. If you agree to this survey, please sign below. Thank you!

Signature of survey respondent (or family member):______________________

No.：______________ Case no.____________ Name:_____

Health Status Questionnaire for Residents of Fujian Province

Hello! In order to understand the health status of the population and the factors affecting it, and to do a good job of disease prevention and health promotion, we are doing some basic surveys on you, and we hope that you will give your support and cooperation, so please recall carefully according to the questions we ask, and please answer truthfully.

I. General information

1.1 Gender: 1=Male 2=Female

1.2 Height ________cm; **Weight** ________kg; **Age** ________

1.3 Date of Birth __________

1.4 Ethnicity**:** 1=Han 2=other ethnic groups, ___________

1.5 Place of birth: ___________ province _____________ city

1.6 What is the highest level of education you have received?

1=Not formally enrolled in school 2=Elementary school 3=Middle school

4=High school (including secondary/technical school) 5=College 6=University

7 = Postgraduate (Masters/Doctorate)

1.7 What is your current occupation __________? Years of work _________

1=Workers 2=Farmers 3=Self-employed 4=Professionals (doctors, teachers, technicians)

5=Unit leader (cadres of organizations, enterprises and institutions)

6=Clerical staff (ordinary civil servants, employees of enterprises and institutions, etc.)

7=Service workers 8=Housewives 9=Retired 10=No occupation

1.8 What is your current marital status?

1=Married 2=Widowed 3=Separated/Divorced 4=Unmarried

1.9 What is the approximate average monthly income (including all sources) for your family?

1= Under ￥3,000 2= ￥3,000-￥6,000 3=￥6,000-9,000 4=￥9,000-￥12,000

5= ￥12,000-￥20,000 6= ￥20,000 or more

1.10 Including yourself, how many members of your family live together? _________person

1.11 What type of health insurance do you currently have?

(1) Basic Medical Insurance for Urban Employee (2) Basic Medical Insurance for Urban Residents

(3) New Rural Cooperative Medical Insurance (4) Commercial Medical Insurance

(5) Government-funded Medical Care (6) Fully Self-paid (7) Unknown

1.12 Have you ever been screened for stomach cancer in the past 5 years? 1=No 2=Yes

1.13 How do you perceive your level of stress in your daily life?

1= High 2= Medium 3= Low 4= No pressure at all

1.14 Prior to diagnosis, did you have a history of stomach surgery? 1=No 2=Yes

1.15 Have you started any of the following treatments? 1=No 2=Yes

⟹ If so, what treatment did you take and when?

☐ Surgery ______________☐ Chemotherapy ______________

☐ Other (please specify) ______________________________

1.16 Have you ever been infected with Helicobacter pylori (H. pylori)?

0 = No 1 = Yes 2 = Not sure

No.: ______________ Case no.____________ Name:_____

II. Behavioral lifestyle

Smoking

(Definition of smoking: one or more cigarettes per day, continuous smoking for more than 6 months, or 150 cigarettes)

2.1 Do you smoke? 0=never 1=yes 2=quit (quit more than 6 months ago)

⟹ **If smoking (including having quit):**

2.1.1 How often do you currently smoke?

1=Occasionally 2=Most days 3=Every day 4=Quit smoking, for_______ years

2.1.2 How many cigarettes do you smoke per day? _______ (currently, or before quitting)

2.1.3 At about what age did you start smoking every day or almost every day? _____ years old

2.2 Have you smoked more than 100 cigarettes in your lifetime? 1=Yes 2=No

2.3 Did your parents smoke when you lived with them?

0=No 1=Only mother smokes 2=Only father smokes 3=Both parents smoke

2.4 As an adult, did the person you live with smoke?

0=No 1=Yes, living together_________years

2.5 Do you currently inhale passive smoke from other smokers?

0= No 1= Occasionally 2= Often

Drinking tea

2.6 How often did you drink tea in the past year?

0 = Hardly ever (<1 time/month) 1 = Occasionally (1-3 times/month)

2 = Rarely (1-2 days/week) 3=Often (3-5 days/week)

4=Almost every day 5=Has quitted **for** _____ years

2.7 At about what age did you start drinking tea every week? ______ years old

☐ I did not drink tea every week

2.8 What kind of tea do you drink regularly (Please choose the most commonly used)

1 = Green tea (Dragon Well tea, Maofeng, Biluochun tea, etc.)

2 = Black Tea (Brick Tea, Jinjunmei black tea, Kung fu tea, etc.)

3 = Oolong tea (Cinnamon, Narcissus, Tieguanyin, Dahongpao, etc.)

4 = Flower tea (rose tea, etc.)

5 = Pu'er Tea

6 = White Tea (White Peony, Shou Mei, etc.)

7 = Other _________

2.9 Do you usually prefer weak tea or strong tea? 1=weak 2=moderate 3=strong ("Strong tea" means that after the tea leaves have been steeped, the volume of tea leaves occupying the cup is more than 50%, "moderate" means that after the tea leaves have been steeped, the volume of tea leaves occupying the cup is more than 25% but less than 50%; "weak tea" means less than 25%.)

2.10 Which tea temperature do you usually prefer? 1=Warm/cool 2=Hot 3=Scalding hot

2.11 What time do you usually drink tea?

1=Before meals 2=After or between meals 3=All situations

Drinking alcohol

2.12 How often did you drink in the past year?

0= Hardly ever (<1 time/month) 1= Occasionally (1-3 times/month)

2= Rarely (1-2 days/week) 3=Often (3-5 days/week)

4=Almost every day 5=Has quitted for _____ years

2.13 At what age did you start drinking every week? ________ years old

☐ I did not drink every week

2.14 What kind of alcohol do you usually drink?

1=Low-alcohol liquor (<40) 2=High-alcohol liquor (≥40°)

3=Fruit wine/rice wine/yellow wine 4=Wine 5=Beer

6=Cocktails (Rio, etc.) 7=Drink all kinds

2.15 On a typical day, how much do you drink? (Please choose the most frequent one.)

1 = Low-alcohol liquor __________ tael/day (____ mL/day)

2 = High-alcohol liquor __________ tael/day (______ mL/day)

3 = Fruit/rice/yellow wine __________ cups/day (______ mL/day)

4 = Wine __________ cups/day (______ mL/day)

5 = Beer __________ bottles/day (______ mL/day)

6 = Cocktail (Rio, etc.) __________ cups/day (______ mL/day)

2.16 When do you usually drink?

1=Drinking with a meal 2=Drinking after or between meals 3=All situations

2.17 Do you experience flushing, rapid heartbeat, and dizziness after drinking?

1=Occurs after one or two sips 2=Occurs after small amounts of alcohol

3=Occurs after large amounts of alcohol 4=No such symptoms occur

Coffee

2.18 Do you usually drink coffee?

0= Hardly ever (<1 time/month) 1= Occasionally (1-3 times/month)

2= Rarely (1-2 days/week) 3=Often (3-5 days/week)

4=Almost every day 5=Has quitted **for** _____ years

2.19 At what age did you start drinking coffee every week? __________ years old

☐ I did not drink coffee every week

2.20 If you drink coffee, how many cups a day do you usually have?

1=1-2 cups/day 2=3-4 cups/day 3=5-6 cups/day 4= More than 6 cups/day

2.21 When do you usually drink coffee?

1=Before a meal 2=After a meal or between meals 3=All situations

III. Dietary habit

Below is a survey of your eating habits **over the past year**.

3.1 Do you have meals regularly: 0 = N o 1=Yes

3.2 What type of rice do you usually consume during meals?

No.: ______________ Case no.____________ Name:______

0= Porridge for all three meals 1= Porridge for two meals

2= Porridge for one meal 3= Do not eat porridge

3.3 Do you prefer hot food: 0=No 1=Occasionally 2=Frequently

3.4 Do you prefer spicy food: 0=No 1=Occasionally 2=Frequently

⟹ **3.4.1 What degree of spicy food you usually prefer?**

1=Do not like spicy food 2=Mildly spicy 3=Moderately spicy 4=Very spicy

⟹ **3.4.2 Frequency of eating spicy food in the past year:**

0= Hardly ever (<1 time/month) 1= Occasionally (1-3 times/month)

2= Rarely (1-2 days/week) 3=Often (3-5 days/week) 4=Almost every day

3.5 Do you enjoy eating hard foods (peanuts, walnuts, chestnuts, beef jerky, etc.)?

0=No 1=Occasionally 2=Frequently

3.6 Do you eat overnight meals:

0=No 1=Occasionally 2=Frequently

3.7 What is your preference for food flavor?

1=Greasy (fried foods) 2=Light 3=Moderate

3.8 Your staple food (choose one):

1=Rice 2=Noodles 3=Corn 4=Sweet potato 5=Other

3.9 What kind of cooking oil do you normally use (choose one commonly used):

1=Peanut oil 2=Animal oil (lard, etc.) 3=Tea oil

4=Soybean/canola oil 5=Blended oil 6=Other

3.10 Are you in the habit of eating the following foods?

**Food group**	**≥4 times /day**	**2-3 times /day**	**1 time /day**	**4-6 times /week**	**2-3 times /week**	**1 time /week**	**1-3 times /month**	**Occasionally**
Rice	≥4 times /day	2-3 times /day	1 time /day	4-6 times /week	2-3 times /week	1 time /week	1-3 times /month	Occasionally
Wheat-based foods	≥4 times /day	2-3 times /day	1 time /day	4-6 times /week	2-3 times /week	1 time /week	1-3 times /month	Occasionally
Whole grains (e.g., corn, sorghum, sweet potatoes)	≥4 times /day	2-3 times /day	1 time /day	4-6 times /week	2-3 times /week	1 time /week	1-3 times /month	Occasionally
Livestock meat and products (e.g., pork, beef, lamb)	≥4 times /day	2-3 times /day	1 time /day	4-6 times /week	2-3 times /week	1 time /week	1-3 times /month	Occasionally
Poultry and products (e.g., chicken, duck)	≥4 times /day	2-3 times /day	1 time /day	4-6 times /week	2-3 times /week	1 time /week	1-3 times /month	Occasionally
Aquatic/seafood products (e.g., fish, shrimp, shellfish)	≥4 times	2-3 times	1 time	4-6 times	2-3 times	1 time	1-3 times	Occasionally

No.：______________ Case no.____________ Name:______

	/day	/day	/day	/week	/week	/week	/month	
Eggs and egg products	≥4 times /day	2-3 times /day	1 time /day	4-6 times /week	2-3 times /week	1 time /week	1-3 times /month	Occasionally
Fresh vegetables	≥4 times /day	2-3 times /day	1 time /day	4-6 times /week	2-3 times /week	1 time /week	1-3 times /month	Occasionally
Fresh fruit	≥4 times /day	2-3 times /day	1 time /day	4-6 times /week	2-3 times /week	1 time /week	1-3 times /month	Occasionally
Soy products (including soymilk)	≥4 times /day	2-3 times /day	1 time /day	4-6 times /week	2-3 times /week	1 time /week	1-3 times /month	Occasionally
Nuts (e.g., peanuts, walnuts, almonds)	≥4 times /day	2-3 times /day	1 time /day	4-6 times /week	2-3 times /week	1 time /week	1-3 times /month	Occasionally
Pickled vegetables and preserved meats	≥4 times /day	2-3 times /day	1 time /day	4-6 times /week	2-3 times /week	1 time /week	1-3 times /month	Occasionally
Dairy products (e.g., milk, yogurt)	≥4 times /day	2-3 times /day	1 time /day	4-6 times /week	2-3 times /week	1 time /week	1-3 times /month	Occasionally
Sweets, pastries, cakes	≥4 times /day	2-3 times /day	1 time /day	4-6 times /week	2-3 times /week	1 time /week	1-3 times /month	Occasionally
Herbal teas and beverages	≥4 times /day	2-3 times /day	1 time /day	4-6 times /week	2-3 times /week	1 time /week	1-3 times /month	Occasionally

3.11 How do you usually handle moldy food (when there are visible spots or stains of mold on the surface)?

0=Discard 1=Clean and eat it 2=Eat directly

3.12 Do you have the habit of having late night snacks?

0= No (skip to 3.13) 1= <1 time/month 2= 1-3 times/month

3= 1-2 times/week 4= 3-5 times/week 5= Almost every day

⟹ **3.12.1 If yes, what time do you have your late night snacks?**

1=22:00-24:00 2=24:00-2:00 3=2:00-4:00

⟹ **3.12.2 If yes, for how many years did you continue to eat late night snacks?____**Years

3.13 In the past year, have you consumed any functional foods that improve gut microbiota and

taken them continuously for more than 30 times?

0=No 1=Yes ⟹_____ times/week and total length of time taken _____ months

(1=Yogurt 2=Prebiotics(益生元) 3=Probiotics(益生菌) 4=Spirulina 5=Other ____________)

IV. Physical activity

(i) Urban residents

6.1 Do you currently live in a town ☐ Yes ☐ No (skip to 6.6)

6.2 In the past year, did you work mainly in a sedentary or standing position or did you mainly do physical work?

1= Predominantly sedentary (e.g., administrators, secretaries, etc.)

2= Standing-oriented (e.g., salesperson, janitor, etc.)

3= Predominantly general physical work

(not too sweaty at room temperature such as plumbing, electrical work, carpentry, masonry, etc.)

4= predominantly heavy manual labor

(sweating easily at room temperature, e.g., loading and unloading, mining, steel making, etc.)

5= Retired or housework, or unemployed for more than one year, or physically disabled and unable to work normally

6.3 How many hours per week do you work on average? ____________ hours

6.4 In the past year, what was your usual method of commuting to and from work or working outside the home? (If yes, go to question 6.5)

1=Walking 2=Riding a motorcycle 3=Riding an electric bicycle

4=Riding a regular bicycle 5=Private/public transportation (car, subway, ferry)

6=Usually work at home/or next to home

6.5 How long is the typical roundtrip time you spend on the road when you commute to and from work (labor outside the home) each day? __________ minutes

(ii) Agricultural workers

6.6 Are you an agricultural laborer☐ Yes☐ No (skip to 6.12)

6.7 On average, how many hours per week do you do farm work, usually/in non-farm busy seasons? _______ hours/week

6.8 Apart from farm work, do you usually do other formal work at the same time? 1=Yes 2=No

6.9 In the other work you do, is the work mainly sedentary or standing or is it mainly heavy manual work?

1 = Predominantly sedentary (e.g., knitting, sewing)

2 = Standing-oriented (e.g., janitors, store clerks)

3= Predominantly general physical labor (e.g., carpenters, electricians, builders, etc.)

4 = Predominantly heavy manual labor (e.g., porters, mining, loading and unloading, etc.)

6.10 How many cumulative hours per week do you typically do other work besides farm work?

___________ hours

6.11 What is the total amount of time you spend on the road on foot or by bicycle each day

No.: ______________ Case no.____________ Name:______

when you go out to work? __________ minutes

(iii) Common parts

6.12 In the past year, how often did you typically participate in physical activity in your spare time?

1=Never or almost never 2=1-3 times per month 3=1-2 times per week

4= 3-5 times per week 5= Exercise every day or almost every day

6.13 If you participate in weekly exercise, which type of exercise do you use most often?

1= Tai chi/qigong/walking 2= Running/aerobics 3= Ball games (basketball, table tennis, badminton, etc.)

4= Brisk walking/health exercises/ranging 5= Swimming 6= Other (e.g. climbing)

6.14 In the past year, how many cumulative hours per week did you participate in physical activity in your spare time? _________ hours/week

6.15 On average, how many cumulative hours per day do you spend doing all types of household chores (including bringing up children)? ________ hours/day

6.16 On average, how many hours per day do you spend in your spare time watching TV/books/newspapers or playing on your cell phone while sitting still? ________ hours/day

V. Food Frequency Questionnaire

**Food**		**In the past year, how often did you eat the following foods**									**Past 10 years if pattern changed**	
Staple food	Rice	≥4 times /day	2-3 times /day	1 time /day	4-6 times /week	2-3 times /week	1 time /week	1-3 times /month	infrequent	not eat	Increase	Decrease
	Congee	≥4 times /day	2-3 times /day	1 time /day	4-6 times /week	2-3 times /week	1 time /week	1-3 times /month	infrequent	not eat	Increase	Decrease
	Noodles (e.g., rice noodles, vermicelli, instant noodles, macaroni, etc.)	≥4 times /day	2-3 times /day	1 time /day	4-6 times /week	2-3 times /week	1 time /week	1-3 times /month	infrequent	not eat	Increase	Decrease
	Steamed buns/stuffed buns	≥4 times /day	2-3 times /day	1 time /day	4-6 times /week	2-3 times /week	1 time /week	1-3 times /month	infrequent	not eat	Increase	Decrease
	Cereal	≥4 times /day	2-3 times /day	1 time /day	4-6 times /week	2-3 times /week	1 time /week	1-3 times /month	infrequent	not eat	Increase	Decrease
	Dumplings	≥4 times /day	2-3 times /day	1 time /day	4-6 times /week	2-3 times /week	1 time /week	1-3 times /month	infrequent	not eat	Increase	Decrease
	Bread	≥4 times /day	2-3 times /day	1 time /day	4-6 times /week	2-3 times /week	1 time /week	1-3 times /month	infrequent	not eat	Increase	Decrease

No.: ______________ Case no.____________ Name:______

	Fried foods (e.g., fried dough cakes, fried dough sticks, fried cakes, sesame cakes, etc.)	≥4 times /day	2-3 times /day	1 time /day	4-6 times /week	2-3 times /week	1 time /week	1-3 times /month	infrequent	not eat	Increase	Decrease
Root vegetables	Sweet potato	≥4 times /day	2-3 times /day	1 time /day	4-6 times /week	2-3 times /week	1 time /week	1-3 times /month	infrequent	not eat	Increase	Decrease
	Taro	≥4 times /day	2-3 times /day	1 time /day	4-6 times /week	2-3 times /week	1 time /week	1-3 times /month	infrequent	not eat	Increase	Decrease
	Potato	≥4 times /day	2-3 times /day	1 time /day	4-6 times /week	2-3 times /week	1 time /week	1-3 times /month	infrequent	not eat	Increase	Decrease
Pickle/ grilled food	Salted duck eggs, century egg	≥4 times /day	2-3 times /day	1 time /day	4-6 times /week	2-3 times /week	1 time /week	1-3 times /month	infrequent	not eat	Increase	Decrease
	Pickled vegetables (e.g., pickled mustard tuber, sauerkraut, pickled radish, pickled bamboo shoots)	≥4 times /day	2-3 times /day	1 time /day	4-6 times /week	2-3 times /week	1 time /week	1-3 times /month	infrequent	not eat	Increase	Decrease
	Fermented tofu, fermented bean paste	≥4 times /day	2-3 times /day	1 time /day	4-6 times /week	2-3 times /week	1 time /week	1-3 times /month	infrequent	not eat	Increase	Decrease
	Pork floss, ham sausage	≥4 times /day	2-3 times /day	1 time /day	4-6 times /week	2-3 times /week	1 time /week	1-3 times /month	infrequent	not eat	Increase	Decrease
Egg	Chicken egg	≥4 times /day	2-3 times /day	1 time /day	4-6 times /week	2-3 times /week	1 time /week	1-3 times /month	infrequent	not eat	Increase	Decrease
	Duck egg	≥4 times /day	2-3 times /day	1 time /day	4-6 times /week	2-3 times /week	1 time /week	1-3 times /month	infrequent	not eat	Increase	Decrease
Fresh meat	Red meat (e.g., pork, beef, lamb)	≥4 times /day	2-3 times /day	1 time /day	4-6 times /week	2-3 times /week	1 time /week	1-3 times /month	infrequent	not eat	Increase	Decrease
	Chicken	≥4 times /day	2-3 times /day	1 time /day	4-6 times /week	2-3 times /week	1 time /week	1-3 times /month	infrequent	not eat	Increase	Decrease
	Duck meat	≥4 times /day	2-3 times /day	1 time /day	4-6 times /week	2-3 times /week	1 time /week	1-3 times /month	infrequent	not eat	Increase	Decrease

No.: ______________ Case no.____________ Name:______

	Pork offal (pork stomach, pork liver, pork blood, etc.)	≥4 times /day	2-3 times /day	1 time /day	4-6 times /week	2-3 times /week	1 time /week	1-3 times /month	infrequent	not eat	Increase	Decrease
	Chicken giblets (gizzards, wings, feet, etc.)	≥4 times /day	2-3 times /day	1 time /day	4-6 times /week	2-3 times /week	1 time /week	1-3 times /month	infrequent	not eat	Increase	Decrease
Seafood	Fish	≥4 times /day	2-3 times /day	1 time /day	4-6 times /week	2-3 times /week	1 time /week	1-3 times /month	infrequent	not eat	Increase	Decrease

Seafood	Shrimp, crab	≥4 times /day	2-3 times /day	1 time /day	4-6 times /week	2-3 times /week	1 time /week	1-3 times /month	infrequent	not eat	Increase	Decrease
	Oyster	≥4 times /day	2-3 times /day	1 time /day	4-6 times /week	2-3 times /week	1 time /week	1-3 times /month	infrequent	not eat	Increase	Decrease
	Kelp	≥4 times /day	2-3 times /day	1 time /day	4-6 times /week	2-3 times /week	1 time /week	1-3 times /month	infrequent	not eat	Increase	Decrease
	Other shellfish (e.g., snails, clams)	≥4 times /day	2-3 times /day	1 time /day	4-6 times /week	2-3 times /week	1 time /week	1-3 times /month	infrequent	not eat	Increase	Decrease
Dairy	Milk	≥4 times /day	2-3 times /day	1 time /day	4-6 times /week	2-3 times /week	1 time /week	1-3 times /month	infrequent	not eat	Increase	Decrease
	Yogurt	≥4 times /day	2-3 times /day	1 time /day	4-6 times /week	2-3 times /week	1 time /week	1-3 times /month	infrequent	not eat	Increase	Decrease
	Soy milk	≥4 times /day	2-3 times /day	1 time /day	4-6 times /week	2-3 times /week	1 time /week	1-3 times /month	infrequent	not eat	Increase	Decrease
	Other milks (goat's milk, camel's milk, etc.)	≥4 times /day	2-3 times /day	1 time /day	4-6 times /week	2-3 times /week	1 time /week	1-3 times /month	infrequent	not eat	Increase	Decrease
Snacks and Dried fruits	Cake	≥4 times /day	2-3 times /day	1 time /day	4-6 times /week	2-3 times /week	1 time /week	1-3 times /month	infrequent	not eat	Increase	Decrease

No.: ______________ Case no.____________ Name:______

(nuts etc)	Biscuits	≥4 times /day	2-3 times /day	1 time /day	4-6 times /week	2-3 times /week	1 time /week	1-3 times /month	infrequent	not eat	Increase	Decrease
	Peanuts	≥4 times /day	2-3 times /day	1 time /day	4-6 times /week	2-3 times /week	1 time /week	1-3 times /month	infrequent	not eat	Increase	Decrease
	Other nuts (cashews, almonds, pistachios, melon seeds, etc.)	≥4 times /day	2-3 times /day	1 time /day	4-6 times /week	2-3 times /week	1 time /week	1-3 times /month	infrequent	not eat	Increase	Decrease
Drinks	Carbonated beverages (Coke, Sprite)	≥4 times /day	2-3 times /day	1 time /day	4-6 times /week	2-3 times /week	1 time /week	1-3 times /month	infrequent	not eat	Increase	Decrease
	Fruit juices (orange juice, etc.)	≥4 times /day	2-3 times /day	1 time /day	4-6 times /week	2-3 times /week	1 time /week	1-3 times /month	infrequent	not eat	Increase	Decrease
	Tea beverages (herbal tea, etc.)	≥4 times /day	2-3 times /day	1 time /day	4-6 times /week	2-3 times /week	1 time /week	1-3 times /month	infrequent	not eat	Increase	Decrease
Soybean product	Soybeans (yellow soybeans, black soybeans, green soybeans)	≥4 times /day	2-3 times /day	1 time /day	4-6 times /week	2-3 times /week	1 time /week	1-3 times /month	infrequent	not eat	Increase	Decrease
	Mung bean	≥4 times /day	2-3 times /day	1 time /day	4-6 times /week	2-3 times /week	1 time /week	1-3 times /month	infrequent	not eat	Increase	Decrease
	Soybean milk	≥4 times /day	2-3 times /day	1 time /day	4-6 times /week	2-3 times /week	1 time /week	1-3 times /month	infrequent	not eat	Increase	Decrease
	Tofu	≥4 times /day	2-3 times /day	1 time /day	4-6 times /week	2-3 times /week	1 time /week	1-3 times /month	infrequent	not eat	Increase	Decrease
	Bean sprouts	≥4 times /day	2-3 times /day	1 time /day	4-6 times /week	2-3 times /week	1 time /week	1-3 times /month	infrequent	not eat	Increase	Decrease
	Other soy products (dried tofu, oil tofu, etc.)	≥4 times /day	2-3 times /day	1 time /day	4-6 times /week	2-3 times /week	1 time /week	1-3 times /month	infrequent	not eat	Increase	Decrease
	Leafy green vegetables (e.g., water spinach, spinach, bok choy, Chinese kale, lettuce)	≥4 times /day	2-3 times /day	1 time /day	4-6 times /week	2-3 times /week	1 time /week	1-3 times /month	infrequent	not eat	Increase	Decrease

No.: ______________ Case no.____________ Name:______

Fresh vegetables	Chinese cabbage	≥4 times /day	2-3 times /day	1 time /day	4-6 times /week	2-3 times /week	1 time /week	1-3 times /month	infrequent	not eat	Increase	Decrease
	Cauliflower	≥4 times /day	2-3 times /day	1 time /day	4-6 times /week	2-3 times /week	1 time /week	1-3 times /month	infrequent	not eat	Increase	Decrease
	Beans (string beans, Dutch beans, string beans)	≥4 times /day	2-3 times /day	1 time /day	4-6 times /week	2-3 times /week	1 time /week	1-3 times /month	infrequent	not eat	Increase	Decrease
	Carrot	≥4 times /day	2-3 times /day	1 time /day	4-6 times /week	2-3 times /week	1 time /week	1-3 times /month	infrequent	not eat	Increase	Decrease
	White radish	≥4 times /day	2-3 times /day	1 time /day	4-6 times /week	2-3 times /week	1 time /week	1-3 times /month	infrequent	not eat	Increase	Decrease
	Celery	≥4 times /day	2-3 times /day	1 time /day	4-6 times /week	2-3 times /week	1 time /week	1-3 times /month	infrequent	not eat	Increase	Decrease
	Onion	≥4 times /day	2-3 times /day	1 time /day	4-6 times /week	2-3 times /week	1 time /week	1-3 times /month	infrequent	not eat	Increase	Decrease
	Ginger	≥4 times /day	2-3 times /day	1 time /day	4-6 times /week	2-3 times /week	1 time /week	1-3 times /month	infrequent	not eat	Increase	Decrease
	Garlic	≥4 times /day	2-3 times /day	1 time /day	4-6 times /week	2-3 times /week	1 time /week	1-3 times /month	infrequent	not eat	Increase	Decrease
	Scallion	≥4 times /day	2-3 times /day	1 time /day	4-6 times /week	2-3 times /week	1 time /week	1-3 times /month	infrequent	not eat	Increase	Decrease
	Cucumbers	≥4 times /day	2-3 times /day	1 time /day	4-6 times /week	2-3 times /week	1 time /week	1-3 times /month	infrequent	not eat	Increase	Decrease
	Other gourds (e.g., loofah, bitter melon, winter melon, pumpkin)	≥4 times /day	2-3 times /day	1 time /day	4-6 times /week	2-3 times /week	1 time /week	1-3 times /month	infrequent	not eat	Increase	Decrease
	Tomato	≥4 times /day	2-3 times /day	1 time /day	4-6 times /week	2-3 times /week	1 time /week	1-3 times /month	infrequent	not eat	Increase	Decrease

No.: ______________ Case no.____________ Name:______

	Chili peppers, green peppers	≥4 times /day	2-3 times /day	1 time /day	4-6 times /week	2-3 times /week	1 time /week	1-3 times /month	infrequent	not eat	Increase	Decrease
	Eggplant	≥4 times /day	2-3 times /day	1 time /day	4-6 times /week	2-3 times /week	1 time /week	1-3 times /month	infrequent	not eat	Increase	Decrease
	Mushrooms (fungus, shiitake, Flammulina velutipes, etc.)	≥4 times /day	2-3 times /day	1 time /day	4-6 times /week	2-3 times /week	1 time /week	1-3 times /month	infrequent	not eat	Increase	Decrease
Fresh fruit	Apple	≥4 times /day	2-3 times /day	1 time /day	4-6 times /week	2-3 times /week	1 time /week	1-3 times /month	infrequent	not eat	Increase	Decrease
	Banana	≥4 times /day	2-3 times /day	1 time /day	4-6 times /week	2-3 times /week	1 time /week	1-3 times /month	infrequent	not eat	Increase	Decrease
	Orange	≥4 times /day	2-3 times /day	1 time /day	4-6 times /week	2-3 times /week	1 time /week	1-3 times /month	infrequent	not eat	Increase	Decrease
	Pear	≥4 times /day	2-3 times /day	1 time /day	4-6 times /week	2-3 times /week	1 time /week	1-3 times /month	infrequent	not eat	Increase	Decrease
	Peach	≥4 times /day	2-3 times /day	1 time /day	4-6 times /week	2-3 times /week	1 time /week	1-3 times /month	infrequent	not eat	Increase	Decrease
	Mango	≥4 times /day	2-3 times /day	1 time /day	4-6 times /week	2-3 times /week	1 time /week	1-3 times /month	infrequent	not eat	Increase	Decrease
	Pineapple	≥4 times /day	2-3 times /day	1 time /day	4-6 times /week	2-3 times /week	1 time /week	1-3 times /month	infrequent	not eat	Increase	Decrease
	Grape	≥4 times /day	2-3 times /day	1 time /day	4-6 times /week	2-3 times /week	1 time /week	1-3 times /month	infrequent	not eat	Increase	Decrease
	Persimmon, persimmon cake	≥4 times /day	2-3 times /day	1 time /day	4-6 times /week	2-3 times /week	1 time /week	1-3 times /month	infrequent	not eat	Increase	Decrease
	Strawberry	≥4 times /day	2-3 times /day	1 time /day	4-6 times /week	2-3 times /week	1 time /week	1-3 times /month	infrequent	not eat	Increase	Decrease

No.：______________ Case no.____________ Name:______

Fresh fruit	Kiwi fruit	≥4 times /day	2-3 times /day	1 time /day	4-6 times /week	2-3 times /week	1 time /week	1-3 times /month	infrequent	not eat	Increase	Decrease
	Pitaya	≥4 times /day	2-3 times /day	1 time /day	4-6 times /week	2-3 times /week	1 time /week	1-3 times /month	infrequent	not eat	Increase	Decrease
Dried food	Shiitake mushrooms (dried)	≥4 times /day	2-3 times /day	1 time /day	4-6 times /week	2-3 times /week	1 time /week	1-3 times /month	infrequent	not eat	Increase	Decrease
	Kelp (dried)	≥4 times /day	2-3 times /day	1 time /day	4-6 times /week	2-3 times /week	1 time /week	1-3 times /month	infrequent	not eat	Increase	Decrease
	Sea weed (dried)	≥4 times /day	2-3 times /day	1 time /day	4-6 times /week	2-3 times /week	1 time /week	1-3 times /month	infrequent	not eat	Increase	Decrease
	Scallops (dried)	≥4 times /day	2-3 times /day	1 time /day	4-6 times /week	2-3 times /week	1 time /week	1-3 times /month	infrequent	not eat	Increase	Decrease
	Fish fillet (dried)	≥4 times /day	2-3 times /day	1 time /day	4-6 times /week	2-3 times /week	1 time /week	1-3 times /month	infrequent	not eat	Increase	Decrease
