# Supplementary material for: Evaluation of the Reliability and Validity of a Food Frequency Questionnaire Using Three-Day 24-Hour Dietary Recalls: A Study in Fujian, China
Source: Nutrients. 2025 Jul 9;17(14):2270. doi: 10.3390/nu17142270 (PMC12298913; doi:10.3390/nu17142270)

Guidelines for Completing the 3-Day 24-Hour Dietary Recall Questionnaire

1. Please record all the food you ate for three consecutive days, Thursday, Friday and Saturday. Fill in one sheet per day for a total of three sheets.

2. Filling method:

The "food name" should be specific, from which you can know the food ingredients or snack trademarks, such as: celery beef dumplings, Yili ice cream, etc.

Baozi and dumplings should be written what the filling is, such as leek and egg baozi; noodles should be written what the broth is or what vegetables are paired with.

"Ingredient composition" refers to the specific ingredients of the food, such as celery beef dumplings. The "food composition" is celery, beef and flour. · "Ingredient weight" refers to the raw weight of each ingredient (that is, the weight of the food before it is cooked), which can be measured in units such as jin, liang, grams (g), milliliters (ml).

"Dining place" is the place where you eat, such as "home", "canteen", "restaurant", "convenience store", etc.

Snacks refer to all foods other than meals. When filling in the form, carefully check the grams and main ingredients on the outer packaging of the food, and calculate the amount of food consumed.
A sample completed questionnaire is illustrated below:

		Food	Raw material composition	Material weight	Where to eat
forenoon	breakfast	pure milk	milk	1 bag (250ml)	home
		Fried leek dumplings	Chives, eggs, flour	Chives 25g, Eggs 50g, Flour 50g	home
afternoon	lunch	rice	rice	200g	Canteen
		Tomato omelette	Tomatoes, eggs	Tomatoes 100g, Eggs 50g	Canteen
		Fried potatoes and carrots	Potatoes, carrots	Potatoes 75g, Carrots 75g	Canteen
		Beef soup noodles	Beef, Noodles	Beef 20g, Noodles 120g	Canteen
	snacks	Oranges	Oranges	100g	home

3. Examples of common food names

Early morning foods:

Soup and porridge: porridge (rice porridge, millet porridge, etc.), fresh milk, milk powder, soy milk (powder), soybean milk, tofu pudding, oatmeal, almond milk, etc.;

Staple food: baozi (leek and pork baozi, celery and beef baozi, etc.), dumplings (celery and pork dumplings, leek and beef dumplings, etc.), fried dough sticks, fried cakes, pancakes, egg pancakes, baked bread, steamed bread, bread, cake, etc.

Lunch and dinner food:

Staple food: rice, porridge, millet porridge, fried rice with eggs, hot soup noodles (noodles with soup), halogenated noodles (noodles + halogen), pancakes, steamed buns, instant noodles, etc.;

Stir-fried dishes: stewed meat: such as carrot and pork, mushroom and chicken,potato and beef, radish and ribs, kelp and ribs; stir-fried meat or eggs: such as green pepper and pork, garlic moss and chicken, celery and beef, cucumber and eggs;

Vegetables: such as stir-fried spinach, home-style tofu, dried tofu (skin), stir-fried potato shreds, stir-fried eggplant, carrot shreds mixed with potato shreds; soups: such as tomato and egg soup, radish bone soup, laver and shrimp skin soup.

Snacks: ice cream, tea drinks, yogurt, biscuits, potato chips, beef jerky, apples, oranges and so on.

Dietary questionnaire (24-hour record): Day 1

		Food	Raw material composition	Material weight	Where to eat
forenoon	breakfast				
	snacks				
afternoon	Lunch				
	snacks				
Night	Dinner				
	snacks				

Dietary questionnaire (24-hour record): Day 2

		Food	Raw material composition	Material weight	Where to eat
forenoon	breakfast				
	snacks				
afternoon	Lunch				
	snacks				
Night	Dinner				
	snacks				

Dietary questionnaire (24-hour record): Day 3

		Food	Raw material composition	Material weight	Where to eat
forenoon	breakfast				
	snacks				
afternoon	Lunch				
	snacks				
Night	Dinner				
	snacks				

100 g mixed-grain rice≈ 1.5 fist-sized portions

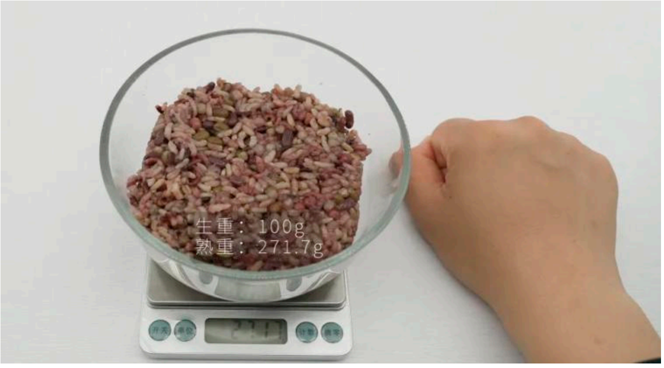

100 g white rice≈ 1 fist-sized portion

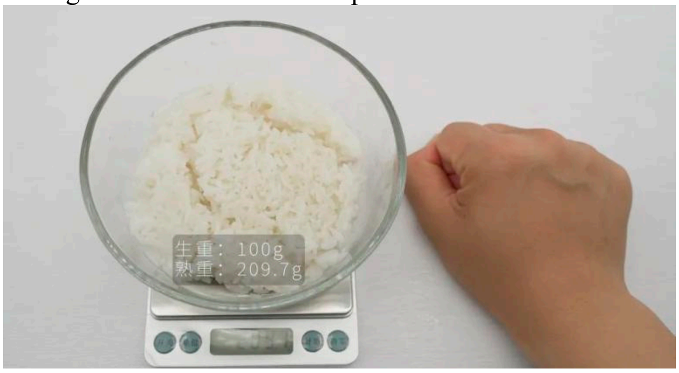

A steamed bun made from 100 g flour≈1 fist-sized portion

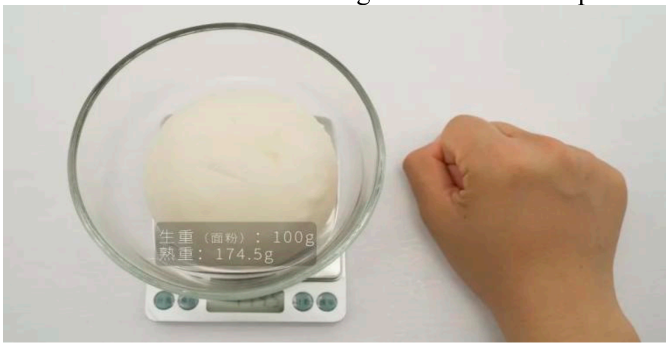

100 g raw sweet potato/yam ≈ half a palm-sized portion

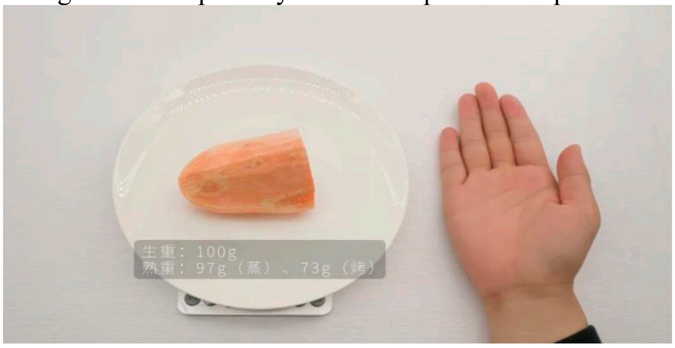

70 g raw chicken breast≈two-thirds of a palm-sized portion

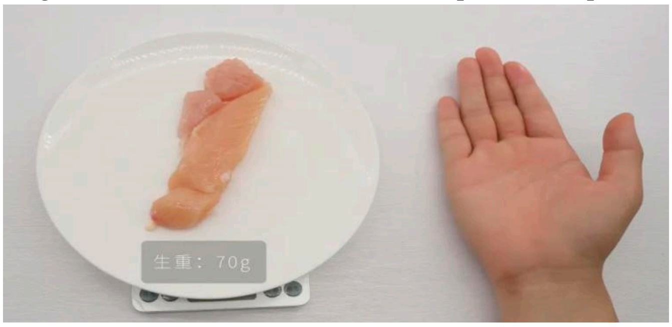

70 g cooked chicken breast≈half a palm-sized portion

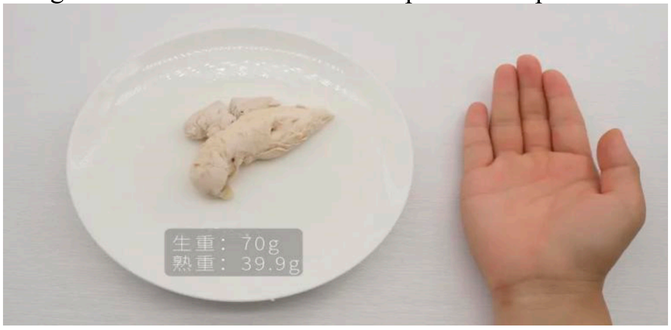

70 g raw shredded lean pork≈1 palm-sized portion

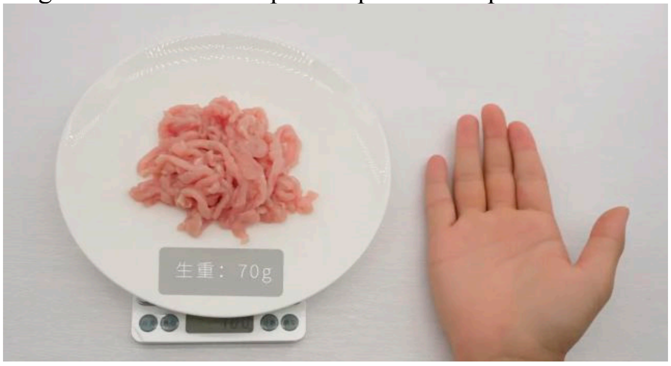

200 g raw shrimp ≈ 2 palm-sized portions

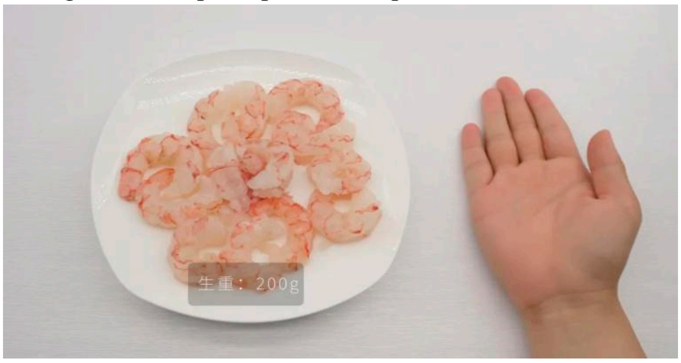

300 g raw fish meat ≈3 palm-sized portions

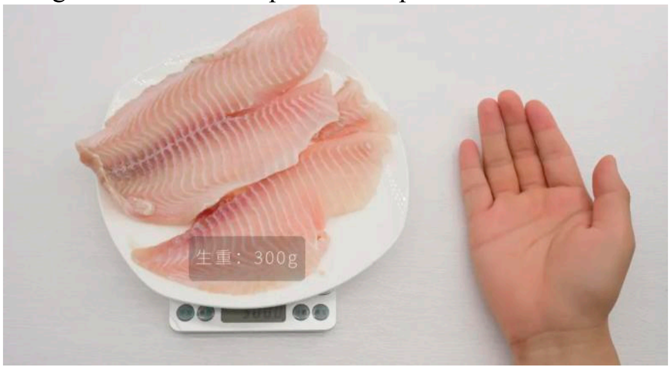

300 g raw cabbage ≈3 palm-sized portions

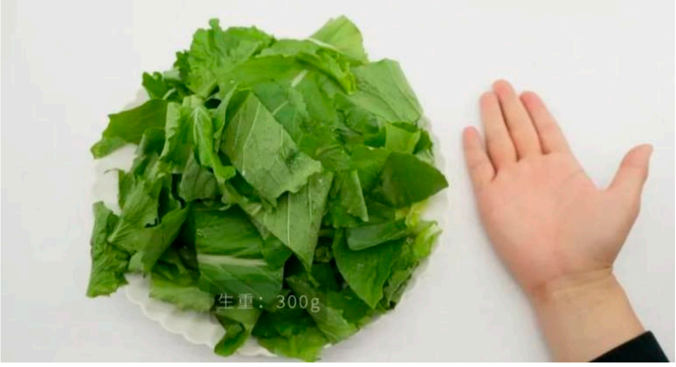

Supplement: Supplementary file 1 [file nutrients-17-02270-s001.zip › Supplemental File S2. 24-hour dietary recall survey.pdf]
