# Supplementary material for: Evaluation of the Reliability and Validity of a Food Frequency Questionnaire Using Three-Day 24-Hour Dietary Recalls: A Study in Fujian, China
Source: Nutrients. 2025 Jul 9;17(14):2270. doi: 10.3390/nu17142270 (PMC12298913; doi:10.3390/nu17142270)
Supplement: Supplementary file 1 [file nutrients-17-02270-s001.zip › Supplemental Tables.pdf]

Table S1 Food group table

Food group	Food items
Refined rice and related products	White rice and rice porridge
Wheat products (e.g., noodles, bread)	Rice noodles, wheat noodles, steamed buns, etc.
Whole grains	Oatmeal and similar cereals
Root and tuber vegetables	Sweet potatoes, taro, potatoes, etc.
Processed meat	Sausages and shredded dried meat
Eggs	Eggs (chicken, duck, etc.)
Red meat	Pork, beef, lamb, etc.
Poultry	Chicken and duck
Organ meat	Pork tripe, pork liver, chicken offal, etc.
Seafood	Fish, shrimp, kelp, etc.
Dairy products	Milk, yogurt, soy milk, etc.
Snacks and nuts	Peanuts, cakes, biscuits/cookies, etc.
Legumes/soy products	Soybeans, mung beans, tofu, etc.
Vegetables	Bok choy, tomatoes, chili peppers, etc.
Fruits	Dragon fruit, apples, bananas, etc.
Beverages	Carbonated drinks, tea-based beverages, juices, etc.

Table S2 Agreement Analysis of Food Group Tertile Categorization Between FFQ-1 and FFQ-2

Food (g/d)	Exact agreement (%)	Adjacent agreement (%)	One-category discrepancy (%)	K_{w*}
Refined rice and related products	66.4	20.1	12.5	0.48
Wheat products (e.g., noodles, bread)	60.5	34.9	4.6	0.50
Whole grains	87.5	12.5	0.0	0.71
Root and tuber vegetables	48.7	46.7	4.6	0.37
Processed meat	56.6	36.4	6.0	0.44
Eggs	69.7	26.3	4.0	0.59

Red meat	58.6	36.2	5.2	0.43
Poultry	73.0	23.1	4.9	0.67
Organ meat	84.2	15.8	0.0	0.60
Seafood	59.9	37.5	2.6	0.50
Dairy products	56.6	39.5	3.9	0.43
Pastries and nuts	55.3	38.2	6.5	0.43
Legumes/soy products	53.3	37.5	9.2	0.38
Vegetables	59.2	36.2	4.6	0.49
Fruits	68.4	24.4	7.2	0.59
Beverages	74.3	18.4	7.3	0.58

*κ_w,Weighted Kappa coefficient

Table S3 Agreement Analysis of Nutrient Intake Tertile Categorization Between FFQ-1 and FFQ-2

Nutrients	Exact agreement (%)	Adjacent agreement (%)	One-category discrepancy (%)	κ_w *
Energy (kcal)	68.4	22.3	9.3	0.56
Protein (g)	55.9	38.9	5.3	0.43
Fat (g)	56.6	39.4	3.9	0.45
Carbohydrates (g)	60.5	35.5	3.9	0.48
Dietary fiber (g)	63.8	34.2	2.0	0.54
Cholesterol (mg)	67.8	30.3	2.0	0.57
Folate (mg)	65.8	32.9	1.4	0.88
Vitamin A (μg)	65.1	31.6	3.3	0.58
Vitamin B1 (mg)	61.8	33.6	4.6	0.54
Vitamin B2 (mg)	65.8	31.6	2.6	0.63
Vitamin B3 (mg)	58.6	38.1	3.3	0.47
Vitamin B6 (mg)	64.5	33.6	2.0	0.66
Vitamin C (mg)	59.2	37.5	3.3	0.50
Vitamin E (mg)	61.8	34.2	3.9	0.51
Calcium (mg)	69.1	27.7	3.3	0.61
Phosphorus (mg)	60.5	34.9	4.6	0.51
Potassium (mg)	58.6	39.4	2.0	0.49
Sodium (mg)	57.9	37.5	4.6	0.49
Magnesium (mg)	69.1	27.0	4.9	0.69
Iron (mg)	53.9	41.5	4.6	0.43
Zinc (mg)	62.5	32.9	4.6	0.53
Selenium (μg)	60.5	34.9	4.6	0.49
Copper (mg)	75.7	20.4	3.9	0.69
Manganese (mg)	74.3	22.3	3.4	0.67

*κ_w,Weighted Kappa coefficient

Table S4 Agreement Analysis of Food Group Tertile Categorization Between FFQ and 24HDR

Food (g/d)	Exact agreement (%)	Adjacent agreement (%)	One-category discrepancy (%)	K_{w*}
Refined rice and related products	51.4	41.6	7.0	0.31
Wheat products (e.g., noodles, bread)	59.9	15.5	5.6	0.50
Whole grains	73.2	5.6	21.2	0.47
Root and tuber vegetables	46.5	48.6	4.9	0.36
Processed meat	56.3	24.6	19.0	0.35
Eggs	47.9	32.4	19.7	0.30
Red meat	51.4	34.5	14.1	0.31
Poultry	51.4	42.3	6.3	0.39
Organ meat	78.2	21.8	0.0	0.44
Seafood	57.7	34.5	7.7	0.45
Dairy products	62.7	30.3	7.0	0.53
Pastries and nuts	61.3	33.1	5.6	0.55
Legumes/soy products	57.0	20.3	12.6	0.40
Vegetables	47.2	40.8	5.0	0.28
Fruits	62.0	33.1	4.9	0.52
Beverages	62.7	21.8	15.5	0.42

*κ_w,Weighted Kappa coefficient

Table S5 Results of Bland-Altman analysis of nutrient intakes between FFQ and 3d-24HDR

Nutrients	Mean difference	95% LOA*
Energy (kcal)	-77.91	-621.78, 465.95
Protein (g)	2.71	-22.48, 27.89
Fat (g)	4.05	-27.42, 35.51
Carbohydrates (g)	2.02	-71.17, 75.21
Dietary fiber (g)	-0.55	-5.07, 3.97
Cholesterol (mg)	-25.03	-429.62, 379.55
Folate (mg)	33.17	-47.38, 113.72
Vitamin A (μg)	20.57	-277.782, 318.93
Vitamin B1 (mg)	-0.08	-0.41, 0.26
Vitamin B2 (mg)	0.07	-0.32, 0.46
Vitamin B3 (mg)	1.13	-7.84, 10.09
Vitamin B6 (mg)	-0.04	-0.29, 0.21
Vitamin C (mg)	20.95	-49.34, 91.23
Vitamin E (mg)	-0.69	-6.11, 4.75

Calcium (mg)	-51.34	-405.66, 302.97
Phosphorus (mg)	53.38	-427.95, 534.71
Potassium (mg)	104.40	-704.51, 913.31
Sodium (mg)	73.89	-351.12, 498.89
Magnesium (mg)	25.05	-97.45, 147.55
Iron (mg)	0.51	-7.51, 8.52
Zinc (mg)	-0.21	-7.68, 7.25
Selenium (μg)	7.92	-33.88, 49.72
Copper (mg)	-0.26	-1.95, 1.44
Manganese (mg)	-0.10	-2.66, 2.45

* LOA: Limits of agreement
